# Editorial: Psycho-Behavioral Factors and Longevity

**DOI:** 10.3389/fpsyg.2022.970345

**Published:** 2022-07-05

**Authors:** Macarena Sánchez-Izquierdo, Hans-Werner Wahl, Oscar Ribeiro, Rocío Fernández-Ballesteros

**Affiliations:** ^1^UNINPSI Clinical Psychology Center, Comillas Pontifical University, Madrid, Spain; ^2^Network Aging Research of Heidelberg University, Heidelberg, Germany; ^3^CINTESIS@RISE, Department of Education and Psychology, University of Aveiro, Aveiro, Portugal; ^4^Department of Psychobiology and Health, Autonomous University of Madrid, Madrid, Spain

**Keywords:** longevity, aging, psycho-behavioral factors, personality, intelligence, emotion, age views, social relations

Longevity has increased since the middle of the nineteenth century and is considered a measure of the success of humanity. An important question that has emerged and various disciplines have attempted to answer is what leads to longevity, in particular to healthy longevity. In this line, two major pathways from a biomedical and demographical perspective are trying to explain why some people live longer and healthier than others: (i) genetic or intrinsic components (i.e., biomedical); and (ii) environmental or extrinsic factors. But another question arises: are these two components alone accountable for longevity and survival?

Presently, it is widely recognized the importance of psycho-behavioral factors in the ways humans age, thus it is necessary to analyse how and to what extent these different psycho-behavioral aspects predict longevity and, above all, healthy longevity. This Research Topic has focused on recent empirical research targeting the variety of psychological factors and linking them to longevity/mortality, thus, to what extent cognition, self-perceptions of aging, perceived control, wellbeing, subjective age, perceived control, social support, or lifestyles, are accounting for longevity. The papers offer various linkages among various structured psycho-behavioral data and objective longevity based on longitudinal data.

Aichele et al. contribute to the Research Topic with the paper “*Cognition-Mortality Associations are More Pronounced when Estimated Jointly in Longitudinal and Time-to-Event Models*,” which compares two-stage analyses vs. joint procedures for estimating longitudinal cognition-mortality associations with and without adjustment for survival-related covariates (smoking and self-rated health). Data for the analyses came from the Manchester Longitudinal Study of Cognition (MLSC). The authors compared these analytic approaches with a repeated-measures study of older adults (ages 50–87 years at assessment). As expected, better cognitive performance predicted lower mortality risk. Importantly, the authors found more prominent cognition-mortality associations from joint analyses compared to two-stage analyses. Associations between mortality risk and crystallized abilities only emerged under joint estimation. The authors highlight the importance of using joint longitudinal survival models for research in cognitive epidemiology.

The paper by Kaspar et al. built on previous research showing that more negative self-perceptions of aging are associated with increased all-cause mortality across rather long observational intervals, even after controlling for relevant confounders. The paper aims to extend this research with the multidimensional construct of awareness of age-related change (AARC) and with a large sample of older adults in their advanced old age (80 years and older) gathered in Germany. Using 3.5 years to estimate determinants of survival, results showed that loss-related AARC indicated the increased risk of survival, whereas gain-related AARC was predictive of longer survival. The effect remained in both cases over and above socio-demographic background characteristics, perceived control, engagement with life, as well as health status.

The paper by Veenstra et al. examined the contribution of psychological factors to mortality patterns in adults aged 67 and older in Norway. All in all, 366 deaths were observed over a mean follow-up of 9.6 years. The study concentrated on baseline subjective age, perceived control, and SES (education and accumulated wealth) as predictors of longevity. Lower levels of perceived control and accumulated wealth independently predict increased mortality, whereas subjective age could not account for additional variability in mortality. Findings underscore that perceived control is an integral component of healthy aging and longevity in addition to socioeconomic inequalities in later life, which play a role in mortality even in a country with an extensive welfare state.

Schilling et al. examined the role of different wellbeing indicators for survival in the oldest-old individuals, using data from the longitudinal project LateLine. The authors address various gaps in the existing literature: (1) they focus on the predictors of survival among very old individuals, (2) analyse the diversity of wellbeing with an integrative examination of multiple indicators of hedonic (i.e., life satisfaction and positive and negative affect) and four indicators of eudaimonic wellbeing (i.e., purpose in life, autonomy, environmental mastery, and self-acceptance); and (3) they include intraindividual changes of wellbeing in the examination of mortality-predictive effects. Through longitudinal multilevel structural equation models, controlling for age, gender, education, and physical condition and testing their sets of hedonic and eudaimonic indictors, the results showed that only one eudaimonic wellbeing indicator, autonomy, showed significant effects on survival.

In addition to these original contributions, two meta-analyses contribute with evidence-based data to the discovery of psycho-behavioral factors, such as lifestyles and social support, which are related to longevity, both throughout life and in advanced age.

Fernández-Ballesteros et al. analyzed in their meta-analysis the association between Behavioral Lifestyles (regular physical activity, healthy diet, sleeping, and weight control) and longevity in the elderly. This meta-analysis included 93 articles, totalling more than 2,800,000 people, and the results highlight the relevance of healthy lifestyles for survival. Furthermore, doing regular physical activity, engaging in leisure activities, sleeping 7–8 h a day, and staying outside the BMI ranges considered as underweight or obesity are habits that each separately has a greater probability associated with survival after several years.

The paper “*Social Support and Longevity: Meta-Analysis-Based Evidence and Psychobiological Mechanisms*” by Vila comprehensively summarized the results of a great extent of published evidence on the predictive value of functional vs. structural measures regarding longevity, and on psychobiological pathways through which social support influences health and longevity. By additionally exposing emergent approaches regarding the link between social support and health (convergent evidence on the effects of social support and adversity in other social mammals; longitudinal studies on the impact of social support and adversity in distinct stages of human lifespan; and research on social support from a community and societal level), the author provides important insights on the research areas that are ought to consolidate the weight social factors have for reduced disease and mortality. The author's synthesis of evidence also calls attention to the need for promoting the culture of social support within large-scale intervention policies.

In conclusion, this Research Topic's compilation of papers significantly increases the evidence underlining that psychological factors matter for longevity. There is an urgent need for future research to better integrate intrinsic, extrinsic, and social-behavioral predictor models of longevity.

## Author Contributions

All authors listed have made a substantial, direct, and intellectual contribution to the work and approved it for publication.

## Conflict of Interest

The authors declare that the research was conducted in the absence of any commercial or financial relationships that could be construed as a potential conflict of interest.

## Publisher's Note

All claims expressed in this article are solely those of the authors and do not necessarily represent those of their affiliated organizations, or those of the publisher, the editors and the reviewers. Any product that may be evaluated in this article, or claim that may be made by its manufacturer, is not guaranteed or endorsed by the publisher.

